# Challenges in heart failure care in four European countries: a comparative study

**DOI:** 10.1093/eurpub/ckad059

**Published:** 2023-05-10

**Authors:** Bianca Steiner, Anne Neumann, Yannick Pelz, Chantal F Ski, Loreena Hill, David R Thompson, Donna Fitzsimons, Lana J Dixon, Julia Brandts, Marlo Verket, Katharina Schütt, Casper G M J Eurlings, Josiane J J Boyne, Arno J Gingele, Lieven De Maesschalck, Marguerite Murphy, Ermelinda Furtado da Luz, Matthew Barrett, Karen Windle, Thom Hoedemakers, Thomas M Helms, Hans-Peter Brunner-La Rocca, Bettina Zippel-Schultz

**Affiliations:** German Foundation for the Chronically Ill, Berlin, Germany; German Foundation for the Chronically Ill, Berlin, Germany; German Foundation for the Chronically Ill, Berlin, Germany; Integrated Care Academy, University of Suffolk, Ipswich, UK; School of Nursing and Midwifery, Queen’s University Belfast, Belfast, UK; School of Nursing and Midwifery, Queen’s University Belfast, Belfast, UK; School of Nursing and Midwifery, Queen’s University Belfast, Belfast, UK; Belfast Health and Social Care Trust, A Floor, Belfast City Hospital, Belfast, UK; Department of Cardiology, University Hospital Aachen, Aachen, Germany; Department of Cardiology, University Hospital Aachen, Aachen, Germany; Department of Cardiology, University Hospital Aachen, Aachen, Germany; Cardiology Department, Laurentius Hospital Roermond, Roermond, The Netherlands; Cardiology Department, Maastricht University Medical Centre, Maastricht, The Netherlands; Department of Health Services Research, CAPHIRI, Maastricht University, Maastricht, The Netherlands; Cardiology Department, Maastricht University Medical Centre, Maastricht, The Netherlands; Thomas More University of Applied Science, Geel, Belgium; Department of Cardiology, St Vincent’s University Hospital, Dublin, Ireland; Department of Cardiology, St Vincent’s University Hospital, Dublin, Ireland; Department of Cardiology, St Vincent’s University Hospital, Dublin, Ireland; Integrated Care Academy, University of Suffolk, Ipswich, UK; Centre for Applied Dementia Studies, Wolfson Centre for Applied Health Research, University of Bradford, Bradford, UK; Sananet Care B.V., Sittard, The Netherlands; German Foundation for the Chronically Ill, Berlin, Germany; Cardiology Department, Maastricht University Medical Centre, Maastricht, The Netherlands; German Foundation for the Chronically Ill, Berlin, Germany

## Abstract

**Background:**

In Europe, more than 15 million people live with heart failure (HF). It imposes an enormous social, organizational and economic burden. As a reaction to impending impact on healthcare provision, different country-specific structures for HF-care have been established. The aim of this report is to provide an overview and compare the HF-care approaches of Germany, Ireland, the Netherlands and the UK, and to open the possibility of learning from each other’s experience.

**Methods:**

A mixed methods approach was implemented that included a literature analysis, interviews and questionnaires with HF-patients and caregivers, and expert interviews with representatives from healthcare, health service research and medical informatics.

**Results:**

The models of HF-care in all countries analyzed are based on the European Society of Cardiology guidelines for diagnosis and treatment of HF. Even though the HF-models differed in design and implementation in practice, key challenges were similar: (i) unequal distribution of care between urban and rural areas, (ii) long waiting times, (iii) unequal access to and provision of healthcare services, (iv) information and communication gaps and (v) inadequate implementation and financing of digital applications.

**Conclusion:**

Although promising approaches exist to structure and improve HF-care, across the four countries, implementation was reluctant to embrace novel methods. A lack of financial resources and insufficient digitalization making it difficult to adopt new concepts. Integration of HF-nurses seems to be an effective way of improving current models of HF-care. Digital solutions offer further opportunities to overcome communication and coordination gaps and to strengthen self-management skills.

## Introduction

Heart failure (HF) is one of the most common cardiovascular diseases worldwide, associated with a high morbidity and mortality.^1^ In Europe, more than 15 million people live with HF.^2^ It is expected that the number of HF-patients will increase in the coming years.^3^ Due to demographic developments, a higher life expectancy, an increasing survival rate of patients with acute cardiac diseases, and advances in HF-diagnostics (resulting in more frequent and earlier detection), the prevalence of HF is increasing, whilst mortality is decreasing due to better therapeutic options.^4^^,^^5^ Diagnosis and treatment of HF are complex and time-consuming, causing high direct and indirect costs, e.g. hospitalizations, medications and loss of labour.^6^^,^^7^ As a chronic disease, HF considerably impairs quality of life. Additionally, HF is often accompanied by comorbidities, such as diabetes.^8^ Subsequently, HF imposes enormous social, organizational and economic burden on patients, their informal caregivers and on healthcare systems.^9^^,^^10^ Different country-specific structures for HF-care have been established in Europe to address these challenges. The report aims to provide an overview of HF-care provision across Germany (DE), Ireland (IRL), the Netherlands (NL) and the UK. The aims of this study are to (1) map the characteristics of the respective healthcare systems, (2) compare the approaches and individual challenges of HF-care and (3) describe the status quo of eHealth and telemedicine implementation.

## Methods

A mixed methods approach was implemented that included a literature analysis and interviews with HF-patients, informal caregivers and interdisciplinary experts.

### Literature analysis

The literature search took place from January to December 2021 by three researchers (A.N., B.S. and Y.P.) with different expertise. To obtain an overview of healthcare systems and map HF-care processes across the countries, information was identified through an open online search of scientific publications, up-to-date electronic newspaper and magazine articles, and (government) reports. Initial searches in PubMed and Google Scholar were supplemented using a snowballing technique. Search terms are composed of the population (patients with HF), the respective country, and the key topics of this report (e.g. diagnosis and management, burden and digital health). Using the information retrieved, search terms were subsequently further refined. In addition to existing reports on individual aspects of HF-care, clinical guidelines were consulted, to identify advised healthcare processes and challenges of HF-care.

### Interview study

Data obtained from an qualitative study originally conducted to elicit requirements and needs of HF-patients and their informal caregivers within the EU-funded project ‘PASSION-HF’ (PAtientSelf-care uSIng eHealth in chrONic Heart Failure; Interreg NWE 702) complemented the analysis.^11^ Between March and June 2019, a total of 49 HF-patients and 33 informal caregivers were interviewed using a semi-structured design.^11^ For recruitment, a maximum variation approach was applied with age, gender, social background and disease severity as key determinants. Ten HF-patients were from DE, 9 from IRL, 18 from NL and 12 from Northern Ireland.^11^ Most were male (76%) aged between 60 and 69 years (43%). Patients and informal caregivers also received questionnaires focusing on healthcare delivery, disease management and attitudes towards technology.^11^ This information was used to integrate insights into the care process and associated challenges from the perspective of patients and their families.

### Expert interviews

Complementary semi-structured interviews were conducted with interdisciplinary experts (DE_n_ = 2, IRL_n_ = 3, NL_n_ = 2, UK_n_ = 2) between December 2021 and January 2022 to clarify and validate country-specific aspects from the literature search. Experts included cardiologists, HF-nurses, health service researchers and medical informatics specialists. Four researchers (A.N., B.S., B.Z. and Y.P.) conducted the interviews. Transcriptions and analyses were presented to the experts in their respective countries for validation.

### Data synthesis

Qualitative and quantitative data were collected and analyzed in a complementary manner. Following a *results-based convergent synthesis design*, data were first analyzed and presented separately.^12^ Subsequently, all information collected on the healthcare systems, care models and digital infrastructures was narratively aggregated in individual country reports, which served as the basis for the comparative analysis. Expert interviews were included sequentially.

## Results

### Characteristics of healthcare systems

Healthcare systems across the four countries differ in their insurance systems, e.g. financial structures and organization of service provision (table 1). While DE has a social insurance system, IRL and UK have a national health service, and NL has a social insurance system with capitation payments.^13^

**Table 1:** Insurance systems in country comparison according to references 13 and 68

	Germany	Ireland	Netherlands	UK
Insurance system (Organization)	Social insurance system Bismarck model	Public healthcare: Beveridge model Private healthcare: private insurers	Social insurance system with capitation fees	Government-sponsored universal healthcare system
Financing	Social insurances (PHI, SHI); contributions from members	Tax financed (+social insurance)	(No distinction)	Tax-financed
Pillars of health insurance	Health insurance Pension insurance Accident insurance Care insurance Unemployment insurance	Basic insurance Private insurance Private supplementary insurance	Basic insurance Care insurance Private supplementary insurance	Basic insurance Optional: private health insurance (faster access to care)
Benefits of basic insurance	Legally regulated services offered by every SHI and thus available to everyone*: GP and specialist treatment, Dental treatment, Medicines, Remedies and medical aids, Inpatient care, Inpatient and outpatient rehabilitation/cure, Normal check-ups and standard vaccinations, Cancer screening tests, Children's sickness benefits, Psychotherapy, Grants to: Dentures Orthodontics for adolescents under 18 years Alternative treatment	Medical card owners: Public outpatient and in-patient services (both mainly in hospitals) Access to cooperating GPs Eye and ears tests Dental checks General: Co-payment levels according to socioeconomic status: Medical card GP visits card Drugs payment scheme card Long-term illness scheme card Treatment of certain infectious diseases Maternity services Services for children	Treatment by general practitioners, specialists, and obstetricians In-patient stays in shared rooms Patient transport Dental services up to the age of 18 Prescription medicines Maternity care Emergency medicine	Medical and dental treatment, Medicines, Remedies and medical aids, Inpatient care, Treatments for childbirth and rehabilitation, Maternity protection and, Preventive benefits

### Models of HF-care

The models of HF-care in all countries are principally based on the European Society of Cardiology (ESC) guidelines for the diagnosis and treatment of acute and chronic HF.^14^ Nevertheless, these models differ in their design and implementation in practice.

Since 2009 evidence-based recommendations have been available in DE with the ‘Nationale Versorgungsleitlinie Chronische Herzinsuffizienz’, relating to outpatient care as well as to aspects of inpatient care.^15^ This focuses on the optimization of therapy to avoid decompensation and hospital admissions and improve coordination of all those involved. In 2018, legal requirements for a Disease Management Program for HF-patients (DMP-HF) were set. They are based on evidence-based guidelines aiming to improve cross-sector treatment and quality of care.^16^ Building on this regulatory framework, insurance companies are allowed to design and offer care programs to patients. However, since the underlying requirements do not reflect medical care reality, the German DMP-HF requires comprehensive revisions and has not yet entered the care process.^17^

In IRL diagnosis and treatment of HF-patients are organized through the national program ‘Heart Failure Model of Care’.^18^ This model aims to organize most aspects of care from prevention to end-of-life care by defining pathways, clinical guidelines and decision support regarding in- and outpatient care. The model also describes the interaction and responsibilities of specific healthcare professionals within the program. The focus is on minimizing hospitalizations by strengthening collaboration between acute care hospitals, general practitioners (GPs) and community care in the sense of an integrated care approach. However, due to lacking investment, especially in primary care, the model has only been implemented in pilot initiatives and is largely limited to a few large urban centres.^19^

In the NL, ESC guidelines were integrated into the national guidelines for GPs in 2010, updated in 2021, and adapted to the Dutch situation to avoid discrepancies between national and regional guidelines mainly used by GPs and international guidelines mainly used by hospital specialist.^20^^,^^21^ To ensure systematic and coordinated treatments, DMPs for HF have been implemented on a national level.^22^ These cover care in hospitals, outpatient clinics or at home.^23^^,^^24^ More recently, the Connect programme aims at implementing agreements across primary, secondary and tertiary care at regional levels to improve HF-care.^25^

In the UK, the National Institute for Health and Care Excellence (NICE) has developed clinical guidelines for the diagnosis and management of HF-patients as quality standards to supplement the ESC guidelines.^26^ NICE guidelines call for interprofessional teamwork—multidisciplinary teams (MDT)—that specialize in HF and work closely with primary care teams. While MDT focus on specialized treatment of HF, the primary care team is responsible for routine patient management, follow-up of HF and ensuring effective communication links between care providers and clinical services involved.

### The role of HF-nurses

**Figure 1:**
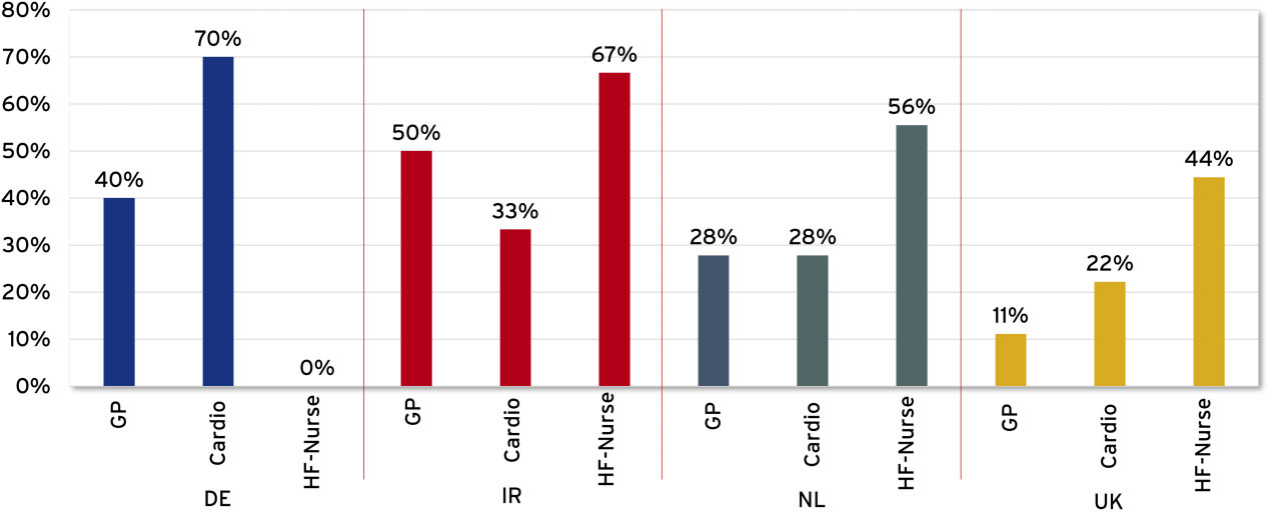
First contact person for patients in case of problems with the heart (multiple answers possible)

In IRL, NL and UK, HF-nurses are usually the first point of contact for patients (figure 1). Despite existing initiatives, such structures have not been established in DE yet.^27^ HF-nurses monitor disease progression and contribute to patient self-management. The role of HF-nurses goes hand-in-hand with a delegation of medical tasks. In NL, specialist nurses have prescribed medication in their area of expertise for more than 10 years.^13^

Although IRL, the NL and the UK have successfully introduced HF-nurses, only NL has managed to ensure adequate provision. In IRL as well as in the UK, the personnel capacities are not sufficient to meet the demand,^19^ resulting in enormous workload for HF-nurses according to the experts. In the UK, for example, HF-nurses report difficulties in patient care due to increases in referrals. Therefore, the British Society for Heart Failure Nurse Forum strongly recommends an increase in the HF-nursing workforce and better integration of community and acute HF-services.^28^

### Regional imbalances

**Figure 2:**
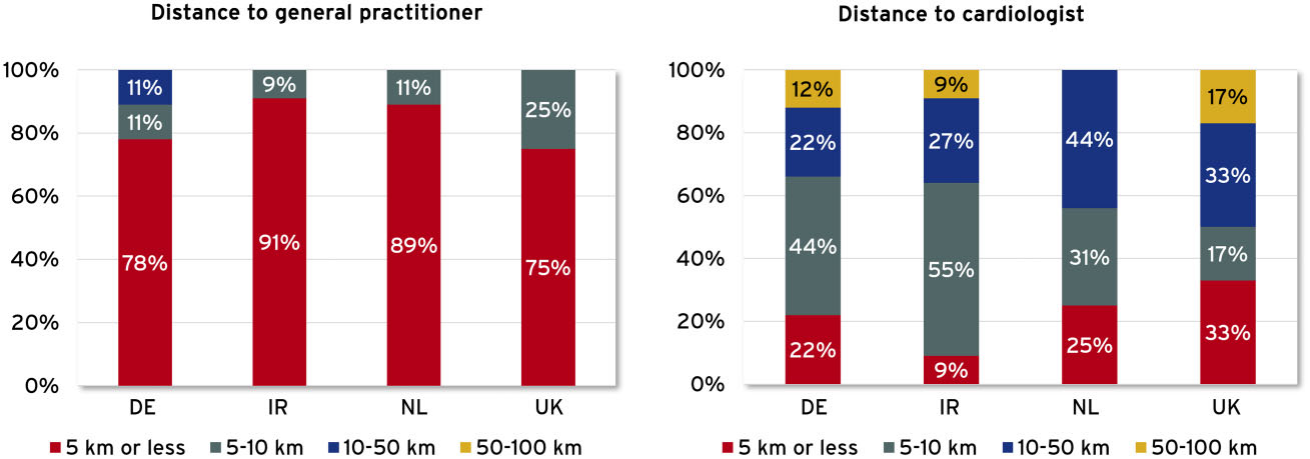
Distance to general practitioners and cardiologist per country

In DE, IRL and parts of the UK, regional differences exist in availability and quality of care provision. While GPs are accessible at closer distances, specialists tend to be concentrated in metropolitan areas, resulting in increased travel time (figure 2). In DE, differences not only exist between urban and rural areas but also between the formerly separated eastern and western regions.^15^ This is due, among other things, to an unbalanced distribution of GPs and specialists not meeting the demand.^29^ Specialists in rural areas have to care for 50–130% more patients than in urban areas.^29^ Federal states and the Associations of Statutory Health Insurance Physicians have therefore launched programmes to attract colleagues to settle in rural areas.^30^

In IRL, the travel distance of patients to GPs is in a range of up to 10 km, the distance to cardiologists was much greater, even up to 50 km (figure 2). According to the experts, the unequal distribution of resources is increased by insufficient funding to implement the national ‘Heart Failure Model of Care’, especially in primary care.^18^ Therefore, current efforts are aimed at providing HF-care as part of a new program for the care of chronically ill and shifting treatment of low-risk patients to the outpatient setting.^18^^,^^31^^,^^32^

NL shows a slightly different picture. While the results of our study show that GPs are accessible at closer distances and that specialists tend to be concentrated in larger and smaller cities, the country has significantly fewer problems with regional differences in access to care, according to the experts. This is due to a high degree of decentralization and good infrastructures.^33^ In NL and UK, specialists, e.g. cardiologists, mostly work in hospital settings.^13^ As in NL, in UK GPs are accessible at closer distances, specialists tend to be concentrated in metropolitan areas.

### Access and supply of healthcare services

In IRL, NL and UK, primary care is mainly provided on an outpatient basis by GPs who act as gatekeepers.^13^ Specialist treatment only takes place after referral. DE has not a classic gatekeeping system.^34^ Patients first consult their GP but may also consult a specialist without receiving a referral.

Long waiting times for specialist care is evident across all countries. The high number of HF-patients meeting a limited number of specialized physicians and nurses, and unequal geographical distributions are the main reasons.^29^^,^^35^^,^^36^ This challenge is particularly visible in IRL. While no more than 4 weeks should pass between referral by GPs and the completion of the diagnosis and therapy plan by a specialist,^31^ the Irish Health Service Executive cites waiting times for referral to a hospital-based outpatient specialist as averaging 6–9 months.^32^ According to our interviews, some patients have to wait for up to 2 years.

In DE, deficits in care also exist regarding an early and valid diagnosis and long-term treatment.^37^ This is partly due to information and communication gaps between GPs, specialists and hospitals but also due to reimbursement. While, for example, an office- or hospital-based cardiologist may perform an echocardiogram (ECG),^15^^,^^38^ GPs rarely perform ECGs or specific blood tests as they are usually not reimbursed for them.^39^

In IRL, further deficits are prominent in rehabilitation services. Although a multidisciplinary rehabilitation should be offered to every patient,^31^ less than 1% are transferred to a cardiac rehabilitation facility.^40^ The Irish Heart Foundation even sees the rehabilitation services in IRL in the middle of an imminent crisis. According to their survey, over 2800 patients are on the national waiting list for cardiac rehabilitation, equalling an increase of 54% since 2013.

In NL, although DMPs for HF have been implemented, differences in supply are apparent in delivery of evidence-based treatments between HF outpatient clinics, e.g. regarding device use.^41^^,^^42^ Less than half of the 6666 patients with reduced left ventricular ejection fraction (LVEF; <50%) received an implantable cardioverter defibrillator or cardiac resynchronization therapy, even when LVEF was below or at 30%.^42^

In the UK, existing deficits in HF-care are mainly due to a high number of patients and limited number of HCPs. Data of the National Heart Failure Audit show that after hospitalization only 55% of HF-patients saw a HF-nurse and only 46% had a cardiology follow-up after discharge.^35^ Experts interviewed, also confirmed that all HF-patients should be seen at least 2–4 times a week for up-titration of evidence-based medication. This is currently not possible.

### Information and communication gaps

All countries face the challenge of overcoming information and communication gaps to ensure a coordinated care process that includes all information to provide high quality care and to avoid multiple examinations and wrong decisions. DE has the hurdle of separated budgets, planning logics, and processes of the inpatient and outpatient sectors and a strong subdivision of medical disciplines.^43^ Those barriers lead to a lack of intersectoral communications and cooperation and subsequently reductions in efficiency and potential misdiagnosis.^44^^,^^45^ According to the experts’ interviews, in IRL, communication problems occur between GPs and HF-nurses, as the nurses in their coordinating role often resort to informal consultations with physicians involved. This complicates adequate documentation due to the lack of a paper trail for the patient’s treatment.

### Implementation of telemedicine and digital health applications

All countries have a national eHealth strategy that is implemented to varying degrees. The existing infrastructure and degree of digitalization in healthcare are particularly high in NL and UK. Both countries already support e-prescriptions and electronic patient records.^46^ However, in NL, since there are more than 3000 different regional electronic patient records connected to the national digital infrastructure AORTA, data exchange between HCPs remains difficult.^47^ In UK, a summary care record is automatically created for patients once they consult a physician that can be viewed by patients. It contains basic information on allergies, vaccinations and current prescriptions. Data are automatically updated as information is added to the practice-based records via their digital infrastructure NHS Spine.

DE and IR are clearly lagging to establish a digital health system.^47^ After several years of developing the national digital infrastructure ‘Telematikinfrastruktur’ in DE, the current infrastructure is considered out of date and is set to be transformed to version 2.0 by 2025.^48^ Despite the shortcoming of digitalization efforts, there are some providers for telemedical solutions in DE—especially in the field of cardiology.^49–51^ Those services (e.g. device monitoring or patient management) are often isolated solutions that are regionally and organizationally separated.^52^ To ensure quality of services, the German Cardiac Society published recommendations that include quality requirements on the staff, organization and evaluation.^53^^,^^54^ Only a few regional telemedicine centres offer their services as part of a certified centre.^49^^,^^55^ The refinancing of digital applications presents a barrier to uptake.^55^ One step forward is that since January 2022, physician practices are refunded for telemonitoring of HF-patients who are treated with a cardiac implant through statutory health insurance (SHI) funds.^56^ Another promising approach is the introduction of Digital Health Applications (DiGAs).^57^^,^^58^ Physicians and psychotherapists can prescribe registered and certified eHealth applications to their patients and bill them via SHIs.^57^ DiGAs are not yet part of routine care.

In IRL, the ‘eHealth Strategy for Ireland’, was published in 2013 focusing on electronic health records, e-prescribing and telemedicine.^59^ IRL also offers some telemedicine applications for HF-patients. This includes a virtual HF-clinic that consists of interactive web conferences where GPs can consult with hospital cardiologists regarding specific patients.^60^ However, there are currently no national digital products that target HF-patients specifically.

In the NL, the government places particular emphasis on applications intended to reduce hospital stays and enable and facilitate outpatient treatment.^46^ The aim is to enable elderly and chronically ill people to be cared for at home for as long as possible. Virtual consultations are in the same way renumerated as face-to-face patient consultations.^61^ Various telemonitoring systems have been developed for patients with heart diseases. Although there is increasing importance of telemonitoring from patients’ perspective in NL,^62^ telemedicine is not yet a widespread reality.^47^ mHealth apps are only offered locally by hospitals or registered physicians and are not subject to a larger framework.^47^ Since January 2022, eHealth applications can be reimbursed by health insurers.

In UK, there are insufficient regulations, standards and protocols for telemedicine.^63^ Currently, some telemonitoring solutions are being tested in pilot projects, but have not yet become an integral part of healthcare provision.^64^ In terms of eHealth apps, however, the UK is further ahead because policymakers recognized the benefits early on.^47^ The most popular eHealth app is the official NHS App, that provides access to certain NHS services like patients’ health records, care plans and appointments.^65^^,^^66^ Some GPs and hospitals offer further services to patients, such as direct messaging or consultation via online forms.^65^

## Discussion

Country-specific structures for HF-care have been established in DE, IRL, NL and UK. Models of HF-care in all countries are principally based on the ESC guidelines for the diagnosis and treatment of HF,^14^ yet they differ in design and implementation. While the gatekeeper model in IRL, NL and UK leads to efficient use of cardiac resources, but carries the risk of delayed specialist care, the German model might result in information gaps and high consumption of resources. However, the challenges are similar and become visible in an unequal distribution of care between urban and rural areas, long waiting times, an inequitable access and supply of healthcare services, information and communication gaps, and insufficient implementation of telemedicine and eHealth. The major challenge is to provide the necessary resources for the increasing number of HF-patients.

A major difference can be seen in the establishment of HF-nurses as focal points in HF-care. If the workforce meets the rising demand, specialist nurses offer a promising approach to facilitate qualitative care. In some cases, they can take over medical tasks such as pharmacological therapy, thus relieving physicians. Successful integration of HF-nurses into care processes can provide much guidance for patients and at the same time relieve the burden on healthcare systems.

The German DiGA concept is unique in Europe and enables reimbursement of specific eHealth applications. However, although the multitude of security and quality standards serve to ensure the development of user-friendly, secure and interoperable applications, they also hamper certification processes. The requirements are difficult to comply with. In addition, only DiGAs classified as risk class I or IIa according to the Medical Device Regulation are eligible. Consequently, the regulation does not apply to many useful applications, such as decision support systems that provide therapy recommendations to empower patients and relieve physicians. Applications for primary prevention are also excluded from reimbursement. Although revision of the legal framework for approval and reimbursement is unavoidable, the DiGA-concept could be a good starting point for further implementation of eHealth solutions in the countries analyzed. A significant contribution could be made to resource-saving structures and self-determined patients by digital solutions.^67^

### Strengths and limitations

This paper provides an overview of the challenges of HF-care across DE, IRL the NL and the UK, considering healthcare systems, care models and digital infrastructures. By highlighting strengths and weaknesses related to the differing HF-models of care, important lessons have been identified. A major challenge in conducting the comparative analysis was the availability of up-to-date data. For some aspects, data availability was very limited, with considerable differences in availability and quality between countries. Overall, information for DE is broader, resulting in a slight emphasis on the German healthcare system. Nevertheless, the explanations provide an impression of the current situation in all analyzed countries and can thus be used as reference.

The findings presented are largely based on associated literature, regulatory documents and medical guidelines, which often describe the optimal or desired status rather than the real situation of care. Electronic newspaper and magazine articles from serious sources were included to reduce bias, adequately describing the actual situation and perceiving critical views. The patient perspective has been integrated into the data synthesis through the interviews conducted in PASSION-HF.^11^

## Conclusions

HF places a high burden on healthcare systems with significant negative social, organizational and economic impacts. Although promising approaches exist in DE, IRL the NL, and the UK to structure and improve HF-care, implementation is lacking and inconsistent. Insufficient digitalization and a lack of financial resources make it difficult to establish new models of care. Notably, integrating HF-nurses has much potential to improve the care situation. However, to be considered, is the issue of resources available for the increasing number of HF-patients. All countries analyzed have some recognition that digital solutions and the introduction of an electronic health record can partly meet the challenges of HF-care, offering further opportunities to overcome communication and coordination gaps and to strengthen self-management of HF-patients. However, the degree of digital implementation varies greatly. Financing of eHealth applications has shown to be difficult.

## Supplementary Material

ckad059_Supplementary_Data

## Data Availability

The datasets generated during and/or analyzed during the current study are available from the corresponding author on reasonable request. In response to the significant social, organizational and economic burden that HF places on patients, families, and healthcare systems, DE, IRL, the NL and the UK have developed different structures for HF-care, each with its own strengths and weaknesses. Access and availability of care vary widely between urban and rural areas in IRL, DE and parts of the UK. Promising approaches to financial structures and to improving care already exist, but implementation is lacking and inconsistent. Integration of HF-nurses seems to be a valid means of improving the care situation. Digital solutions offer another opportunity to overcome communication and coordination deficits and strengthen self-management skills.

## Notes

The references 41 to 68 are available in Supplementary material.
