# Supplementary material for: Challenges in heart failure care in four European countries: a comparative study
Source: Eur J Public Health. 2023 May 10;33(3):448–54. doi: 10.1093/eurpub/ckad059 (PMC10234648; doi:10.1093/eurpub/ckad059)
Supplement: ckad059_Supplementary_Data [file ckad059_supplementary_data.docx]

# Full list of references

1. Savarese G, Lund LH. Global Public Health Burden of Heart Failure. Card Fail Rev. 2017;3(1):7-11.

2. Heart Failure Policy Network. Heart failure policy and practice in Europe. London: HFPN; 2020. Available from: http://www.healthpolicypartnership.com/app/uploads/Heart-failure-policy-and-practice-in-Europe.pdf.

3. Bundesärztekammer, Kassenärztliche Bundesvereinigung, Arbeitsgemeinschaft der Wissenschaftlichen Medizinischen Fachgesellschaften. Nationale VersorgungsLeitlinie: Chronische Herzinsuffizienz (Langfassung) 2019. Available from: https://www.kbv.de/media/sp/nvl-herzinsuffizienz-lang.pdf.

4. Gomez-Soto FM, Andrey JL, Garcia-Egido AA, Escobar MA, Romero SP, Garcia-Arjona R, et al. Incidence and mortality of heart failure: a community-based study. Int J Cardiol. 2011;151(1):40-5.

5. McMurray JJV, Stewart S. The burden of heart failure. Eur Heart J S. 2002;4(suppl_D):D50-D8.

6. Lesyuk W, Kriza C, Kolominsky-Rabas P. Cost-of-illness studies in heart failure: a systematic review 2004–2016. BMC Cardiovasc Disord. 2018;18(1):1-11.

7. Lonn E, McKelvie R. Drug treatment in heart failure. BMJ. 2000;320(7243):1188-92.

8. van der Wal HH, van Deursen VM, van der Meer P, Voors AA. Comorbidities in Heart Failure. Handb Exp Pharmacol. 2017;243:35-66.

9. Cook C, Cole G, Asaria P, Jabbour R, Francis DP. The annual global economic burden of heart failure. Int J Cardiol. 2014;171(3):368-76.

10. Lesyuk W, Kriza C, Kolominsky-Rabas P. Cost-of-illness studies in heart failure: a systematic review 2004–2016. BMC Cardiovasc Disord. 2018;18(1).

11. Zippel-Schultz B, Palant A, Eurlings C, F Ski C, Hill L, Thompson DR, et al. Determinants of acceptance of patients with heart failure and their informal caregivers regarding an interactive decision-making system: a qualitative study. BMJ Open. 2021;11(6):e046160.

12. Noyes J, Booth A, Moore G, Flemming K, Tunçalp Ö, Shakibazadeh E. Synthesising quantitative and qualitative evidence to inform guidelines on complex interventions: clarifying the purposes, designs and outlining some methods. BMJ Glob Health. 2019;4(Suppl 1):e000893.

13. Schölkopf M, Grimmeisen S. Das Gesundheitswesen im internationalen Vergleich. Gesundheitssystemvergleich, Länderberichte und europäische Gesundheitspolitik. Berlin: MWV Medizinisch Wissenschatliche Verlagsgesellschaft mbH & Co. KG; 2020.

14. McDonagh TA, Metra M, Adamo M, Gardner RS, Baumbach A, Böhm M, et al. 2021 ESC Guidelines for the diagnosis and treatment of acute and chronic heart failure. Eur Heart J. 2021;42(36):3599-726.

15. Riens B, Bätzing-Feigenbaum J. Leitliniengerechte Therapie bei Herzinsuffizienz2014 26.10.2021.

16. Strukturierte Behandlung der Herzinsuffizienz künftig in eigenständigem Disease-Management-Programm [press release]. Berlin, 19.04.2018 2018.

17. Deutsches Ärzteblatt. IQWiG sieht Aktualisierungs­bedarf beim DMP Herzinsuffizienz 2021 [updated 26.07.2021. Available from: https://www.aerzteblatt.de/nachrichten/125885/IQWiG-sieht-Aktualisierungsbedarf-beim-DMP-Herzinsuffizienz.

18. Health Service Executive. National heart failure clinical care program: heart failure model of care 2012 [Available from: https://www.hse.ie/eng/services/publications/clinical-strategy-and-programmes/heart-failure-model-of-care-jan-2012.pdf.

19. The Heart Failure Policy Network. Heart failure policy and practice in Europe: Ireland: The Health Policy Partnership Ltd.; 2020. Available from: https://www.hfpolicynetwork.org/wp-content/uploads/Heart-failure-policy-and-practice-in-Europe-Ireland.pdf.

20. Kasje WN, Denig P, De Graeff PA, Haaijer-Ruskamp FM. Physicians' views on joint treatment guidelines for primary and secondary care. Int J Qual Health Care. 2004;16(3):229-36.

21. Nederlands Huisartsen Genootschap. NHG-Richtlijnen. Hartfalen. 2010 [Available from: https://richtlijnen.nhg.org/multidisciplinaire-richtlijnen/hartfalen.

22. Meems LMG, van Veldhuisen DJ, de Boer RA. Progress in heart failure management in the Netherlands and beyond: long-term commitment to deliver high-quality research and patient care. Neth Heart J. 2020;28(Suppl 1):31-8.

23. de Loor S, Jaarsma T. Nurse-managed heart failure programmes in the Netherlands. Eur J Cardiovasc Nurs. 2002;1(2):123-9.

24. Jaarsma T, Tan B, Bos RJ, van Veldhuisen DJ. Heart failure clinics in the Netherlands in 2003. Eur J Cardiovasc Nurs. 2004;3(4):271-4.

25. NVVC Connect. Juiste Hartzorg Op de Juiste Plek 2022 [Available from: https://www.nvvcconnect.nl/.

26. National Institute for Health and Care Excellence. Chronic heart failure in adults: diagnosis and management 2018. Available from: https://www.nice.org.uk/guidance/ng106.

27. Störk S, Kindermann I, Jacobs M, Perings S, Raake P, Rosenkranz S, et al. Fortbildungscurriculum: Spezialisierte Herzinsuffizienz-Assistenz. Aktuelle Kardiologie. 2020;9(01):90-5.

28. Masters J, Barton C, Blue L, Welstand D. Increasing the heart failure nursing workforce: recommendations by the British Society for Heart Failure Nurse Forum. Br J Card Nurs. 2019;14(11):1-12.

29. Schwenk U. Ärztedichte - Neue Bedarfsplanung geht am Bedarf vorbei. Bertelsmann Stiftung, editor. Gütersloh: Bertelsmann Stiftung; 2015. 8 p.

30. Niedersächsisches Ministerium für Soziales, Gesundheit und Gleichstellung. Evaluation der Maßnahmen zur Sicherung der ärztlichen Versorgung auf dem Land in Niedersachsen2019 11.07.2022. Available from: https://www.ms.niedersachsen.de/download/150192.

31. Clinical Strategy and Programmes Directorate, HSE. Heart Failure Model of Care [National Heart Failure Clinical Care Programme]. 2012.

32. Health Service Executive. Terms of Agreement between the Department of Health, the HSE and the IMO regarding GP Contractual Reform and Service Development2019 11.07.2022]. Available from: https://www.hse.ie/eng/about/who/gmscontracts/2019agreement/agreement-2019.pdf.

33. Schoen C, Osborn R, Squires D, Doty M, Pierson R, Applebaum S. New 2011 Survey Of Patients With Complex Care Needs In Eleven Countries Finds That Care Is Often Poorly Coordinated. Health Affairs. 2011;30(12):2437-48.

34. Hoebel J, Rattay P, Prütz F, Rommel A, Lampert T. Socioeconomic Status and Use of Outpatient Medical Care: The Case of Germany. PLOS ONE. 2016;11(5):e0155982.

35. National Institute for Cardiovascular Outcomes Research (NICOR), British Society for Heart Failure (BSH), Barts Health NHS Trust, The Healthcare Quality Improvement Partnership (HQIP). National Heart Failure Audit (NHFA). 2021 Summary Report 2021 [Available from: https://www.nicor.org.uk/wp-content/uploads/2021/10/NHFA-Domain-Report_2021_FINAL.pdf

36. OECD & European Observatory on Health Systems and Policies. The Netherlands. Country Health Profile 2019: OECD Publishing; 2019.

37. Störk S, Peters-Klimm F, Bleek J, Ninic R, Klöss A. Sektorübergreifende Versorgung bei Herzinsuffizienz. Springer Berlin Heidelberg; 2021. p. 109-30.

38. Deutsche Herzstiftung. Deutscher Herzbericht 2020. Frankfurt am Main: Thieme; 2021. Available from: https://www.herzstiftung.de/system/files/2021-06/Deutscher-Herzbericht-2020.pdf.

39. Störk S, Handrock R, Jacob J, Walker J, Calado F, Lahoz R, et al. Treatment of chronic heart failure in Germany: a retrospective database study. Clin Res Cardiol. 2017;106(11):923-32.

40. The Heartbeat Trust, Irish Heart Foundation, NUI Galway, Novartis Pharmaceutiucals. The Cost of Heart Failure in Ireland - The social, economic and health implications of Heart Failure in Ireland 2015. Available from: https://www.rte.ie/documents/news/cost-of-heart-failure-report-web.pdf.

41. Brunner-La Rocca HP, Linssen GC, Smeele FJ, van Drimmelen AA, Schaafsma HJ, Westendorp PH, et al. Contemporary Drug Treatment of Chronic Heart Failure With Reduced Ejection Fraction: The CHECK-HF Registry. JACC Heart Fail. 2019;7(1):13-21.

42. Raafs AG, Linssen GCM, Brugts JJ, Erol-Yilmaz A, Plomp J, Smits JPP, et al. Contemporary use of devices in chronic heart failure in the Netherlands. ESC Heart Fail. 2020;7(4):1771-80.

43. Breckenkamp J, Wiskow C, Laaser U. Progress on quality management in the German health system – a long and winding road. Health Research Policy and Systems. 2007;5(1):7.

44. Moore C, Wisnivesky J, Williams S, McGinn T. Medical errors related to discontinuity of care from an inpatient to an outpatient setting. J Gen Intern Med. 2003;18(8):646-51.

45. Gowda NR, Kumar A, Arya SK, H V. The information imperative: to study the impact of informational discontinuity on clinical decision making among doctors. BMC Med Inform Decis Mak. 2020;20(1):175.

46. Bundesministerium für Wirtschaft und Energie. Niederlande: Digitalisierung im Gesundheitswesen. Den Haag: Deutsch-Niederländische Handelskammer; 2021.

47. Thiel R, Deimel L, Schmidtmann D, Piesche K, Hüsing T, Rennoch J, et al. #SmartHealthSystems Digitalisierungsstrategien im internationalen Vergleich. Gütersloh: Bertelsmann Stiftung; 2018. Available from: https://www.bertelsmann-stiftung.de/de/publikationen/publikation/did/smarthealthsystems/.

48. gematik. An arena for digital health: Telematics Infrastructure 2.0 white paper for a federally networked healthcare system. Berlin: gematik GmbH; 2021. Available from: https://www.gematik.de/fileadmin/user_upload/gematik/files/Publikationen/gematik_Arena_for_digital_health_white_paper_TI_2.0_web_EN_202101.pdf.

49. Van den Berg N, Schmidt S, Stentzel U, Mühlan H, Hoffmann W. Telemedizinische Versorgungskonzepte in der regionalen Versorgung ländlicher Gebiete. Bundesgesundheitsblatt-Gesundheitsforschung-Gesundheitsschutz. 2015;58(4-5):367-73.

50. Koehler F, Koehler K, Deckwart O, Prescher S, Wegscheider K, Kirwan B-A, et al. Efficacy of telemedical interventional management in patients with heart failure (TIM-HF2): a randomised, controlled, parallel-group, unmasked trial. Lancet. 2018;392(10152):1047-57.

51. gematik. Übersicht aller Projekte & Anwendungen. vesta Informationsportal; 2021.

52. Thiel R, Deimel L. Einsatz und Nutzung von Telemedizin – Länderüberblick. Gütersloh: Bertelsmann Stiftung; 2020. Available from: https://www.bertelsmann-stiftung.de/fileadmin/files/BSt/Publikationen/GrauePublikationen/VV_SHS_Telemedizin.pdf.

53. Helms T, Stockburger M, Köhler F, Leonhardt V, Müller A, Rybak K, et al. Grundlegende Strukturmerkmale eines kardiologischen Telemedizinzentrums für Patienten mit Herzinsuffizienz und implantierten Devices, Herzrhythmusstörungen und erhöhtem Risiko für den plötzlichen Herztod. Herzschrittmachertherapie + Elektrophysiologie. 2019;30(1):136-42.

54. Helms TM, Köpnick A, Perings CA, Dürsch M, Leonhardt V, Pauschinger M, et al. Positionspapier zum Anforderungsprofil von nichtärztlichen Assistenzkräften in Telemedizinzentren. Herzschrittmachertherapie + Elektrophysiologie. 2021;32(4):504-9.

55. Secer S, von Bandemer S. Potenziale und Perspektiven der Telemedizin. Forschung Aktuell [Internet]. 2019; 03/2019. Available from: https://www.econstor.eu/bitstream/10419/193767/1/1067244328.pdf.

56. Deutsches Ärzteblatt. Telemonitoring zu Herzinsuffizienz kann starten. Ärzteschaft [Internet]. 2022 31.01.2022. Available from: https://www.aerzteblatt.de/nachrichten/131226/Telemonitoring-zu-Herzinsuffizienz-kann-starten.

57. Federal Institute for Drugs and Medical Devices. Das Fast Track Verfahren für digitale Gesundheitsanwendungen (DiGA) nach § 139e SGB V. Bonn: Bundesinstitut für Arzneimittel und Medizinprodukte (BfArM); 2020. Available from: https://www.bfarm.de/SharedDocs/Downloads/DE/Medizinprodukte/diga_leitfaden.pdf?__blob=publicationFile.

58. Federal Ministry of Health. Digitale-Gesundheitsanwendungen-Verordnung (DiGAV) 2022 [updated 11.01.2022. Available from: https://www.bundesgesundheitsministerium.de/service/gesetze-und-verordnungen/guv-19-lp/digav.html.

59. Health Information and Quality Authority. eHealth in Ireland: Frequently asked questions. Dublin: Health Information and Quality Authority,; 2021. Available from: https://www.hiqa.ie/sites/default/files/2021-01/eHealth-in-Ireland_FAQ_0.pdf.

60. An Roinn Slàinte, Irsih Medical Organisation, Health Service Executive. Terms of Agreement between the Department of Health, the HSE and the IMO regarding GP Contractual Reform and Service Development 2019. Available from: https://www.hse.ie/eng/about/who/gmscontracts/2019agreement/agreement-2019.pdf.

61. Ministry of Health WaS. Government encouraging the use of eHealth (telehealth) 2021 [Available from: https://www.government.nl/topics/ehealth/government-encouraging-use-of-ehealth.

62. Wouters M, Huygens MWJ, Voogdt H, Meurs MM, de Groot J, Lamain A, et al. Samen aan zet! den Haag, Utrecht: Nictiz Nivel; 2019. Available from: https://www.nictiz.nl/wp-content/uploads/eHealth19_Rapport.pdf.

63. Morgan Lewis. Telehealth in the United Kingdom: Considerations for Providers 2021 [Available from: https://www.morganlewis.com/pubs/2021/02/telehealth-in-the-united-kingdom-considerations-for-providers-cv19-lf.

64. Northern Health and Social Care Trust. Remote Telemonitoring Antrim2020 [Available from: http://www.northerntrust.hscni.net/services/remote-telemonitoringtelehealthtelecare/.

65. NHS Digital. About the NHS App 2021 [updated 20.12.2021. Available from: https://www.nhs.uk/nhs-app/about-the-nhs-app/.

66. NHS Digital. NHS App 2021 [updated 15.12.2022. Available from: https://digital.nhs.uk/services/nhs-app.

67. Helms TM, Köpnick A, Leber A, Zugck C, Steen H, Karle C, et al. Herzinsuffizienzversorgung in einer digitalisierten Zukunft. Der Internist. 2021;62(11):1180-90.

68. Geseztlichekrankenkassen.de. Gesetzliche Pflichtleistungen in 2022 2022 [11.07.2022]. Available from: https://www.gesetzlichekrankenkassen.de/leistungen/leistungen.html.
